# Suppression of IL-12p70 formation by IL-2 or following macrophage depletion causes T-cell autoreactivity leading to CNS demyelination in HSV-1-infected mice

**DOI:** 10.1371/journal.ppat.1006401

**Published:** 2017-05-22

**Authors:** Dhong Hyun Lee, Mandana Zandian, Jane Kuo, Kevin R. Mott, Shuang Chen, Moshe Arditi, Homayon Ghiasi

**Affiliations:** 1 Center for Neurobiology and Vaccine Development, Ophthalmology Research, Department of Surgery, Cedars-Sinai Burns & Allen Research Institute, CSMC – SSB3, Los Angeles, California, United States of America; 2 Division of Pediatric Infectious Diseases and Immunology, CSMC, Los Angeles, California, United States of America; University of Southern California, UNITED STATES

## Abstract

We have established two mouse models of central nervous system (CNS) demyelination that differ from most other available models of multiple sclerosis (MS) in that they represent a mixture of viral and immune triggers. In the first model, ocular infection of different strains of mice with a recombinant HSV-1 that expresses murine IL-2 constitutively (HSV-IL-2) causes CNS demyelination. In the second model, depletion of macrophages causes CNS demyelination in mice that are ocularly infected with wild-type (WT) HSV-1. In the present study, we found that the demyelination in macrophage-intact mice infected with HSV-IL-2 was blocked by depletion of FoxP3-expressing cells, while concurrent depletion of macrophages restored demyelination. In contrast, demyelination was blocked in the macrophage-depleted mice infected with wild-type HSV-1 following depletion of FoxP3-expressing cells. In macrophage-depleted HSV-IL-2-infected mice, demyelination was associated with the activity of both CD4^+^ and CD8^+^ T cells, whereas in macrophage-depleted mice infected with WT HSV-1, demyelination was associated with CD4^+^ T cells. Macrophage depletion or infection with HSV-IL-2 caused an imbalance of T cells and T_H_1 responses as well as alterations in IL-12p35 and IL-12p40 but not other members of the IL-12 family or their receptors. Demyelination was blocked by adoptive transfer of macrophages that were infected with HSV-IL-12p70 or HSV-IL-12p40 but not by HSV-IL-12p35. These results indicate that suppression of IL-12p70 formation by IL-2 or following macrophage depletion causes T-cell autoreactivity leading to CNS demyelination in HSV-1-infected mice.

## Author summary

Several mouse models of multiple sclerosis (MS) are now available. We have established two new mouse models. In the first model, ocular infection of different strains of mice with HSV-IL-2 recombinant virus causes CNS demyelination. In the second model, CNS demyelination was induced by different strains of wild type HSV-1 in the absence of macrophages. In the present study, we found differences in T-cell reactivity in the two models. However, both models exhibited an imbalance in IL-12p35 and IL-12p40. The requirement for formation of the IL-12p70 dimer in prevention of demyelination was supported by adoptive transfer experiments. These results suggest a pathological role for macrophages in these models of virus-induced MS in which suppression of IL-12p70 formation by IL-2 or following macrophage depletion causes T-cell autoreactivity leading to CNS demyelination.

## Introduction

Multiple sclerosis (MS) is due to degradation of the myelin sheath [1] and visual disorders due to demyelination of the optic nerve is the early sign of individuals diagnosed with MS [2,3]. Thus, optic neuritis can be used as an early factor for detection of MS. Both genetic and environmental factors are implicated in development of optic neuritis and MS [4–8]. Considerable evidence supports the concept that dysregulation of IL-2 plays a critical role in the development of MS [9–18]. We therefore developed a model of MS in which we combined altered expression of IL-2 with an environmental signal, HSV-1 infection. In this model, ocular infection of mice with HSV-IL-2 recombinant virus caused demyelination in the brain, spinal cord, and optic nerve [19,20]. Ocular infection with parental, wild-type (WT) viruses, or with recombinant HSV-1 expressing either IFN-γ or IL-4, did not induce CNS demyelination. Similar results were obtained following delivery of rIL-2 protein, IL-2 DNA or IL-2 synthetic peptides prior to infection with different strains of HSV-1 [21]. Thus, the HSV-IL-2 offers a new and different small animal model for MS that integrates an environmental (viral) signal [19,20,22–24]. In this HSV-IL-2 model, the production of IL-2 by HSV-IL-2 is similar to the increases in IL-2 that have been observed in MS and there was increased T-cell autoreactivity leading to the CNS demyelination.

The second model arose from the finding that ocular infection of macrophage-depleted mice with WT HSV-1 leads to demyelination in the absence of an external source of IL-2. CNS demyelination did not occur in macrophage-intact mice that were ocularly infected with WT HSV-1 in the absence of an external source of IL-2 [19,20,22–24] and CNS demyelination did not occur on depletion of T cells, B cells, dendritic cells (DCs), or natural killer (NK) cells following ocular infection with WT HSV-1 [22].

The identification of these two closely related models provided the opportunity to use a comparative analysis approach to identify the mechanisms by which macrophages may contribute to, or modulate, demyelination in the context of ocular viral infection. Macrophages are mononuclear phagocytes that play critical roles in development, tissue homeostasis and the resolution of inflammation [25]. The wide variety of functions exhibited by macrophages include cytokine secretion and antigen presentation, and cytotoxicity as well as phagocytosis. Macrophage infiltrates are an integral component of the immune defense system. They are central to innate immunity and contribute to the intersection between innate and adaptive immunity. A number of factors are known to "activate" or engage macrophages in these activities, including viral infection. Following infection of naive mice with HSV-1, macrophages are the major infiltrates of the eye [26–28], and play a central role in both enhancement and blocking of inflammation in the eye [29–33]. In addition to their phagocytosis, antigen presentation and cytokine production [34,35], macrophages are the major source of IL-12 production [36,37], a cytokine reported to be involved in stimulation of both T cells and NK cells [38–40]. IL-12 also has been shown to enhance the T_H_1 response [41–43].

The results of our current study demonstrate that: 1) In the HSV-IL-2 model, demyelination was eliminated on depletion of FoxP3-expressing cells, when macrophages were present but not when macrophages were depleted. In contrast, the macrophage-depleted HSV-1 infected mice did not show demyelination when Foxp3-expressing cells were depleted. However, in the absence of macrophages FoxP3^-^ T cells caused demyelination; 2) In both models, macrophages played a critical role in prevention of autoimmunity; 3) Either suppression of macrophages by IL-2 or their absence caused an imbalance of T cells and the development of autoaggressive T cells; and 4) Adoptive transfer of macrophages over-expressing IL-12p70 or IL-12p40, but not IL-12p35, blocked HSV-1 induced CNS demyelination in a dose-dependent manner. Collectively, our results suggest that macrophages play a major role in protection against HSV-1-induced CNS demyelination.

## Results

**Fig 1:**
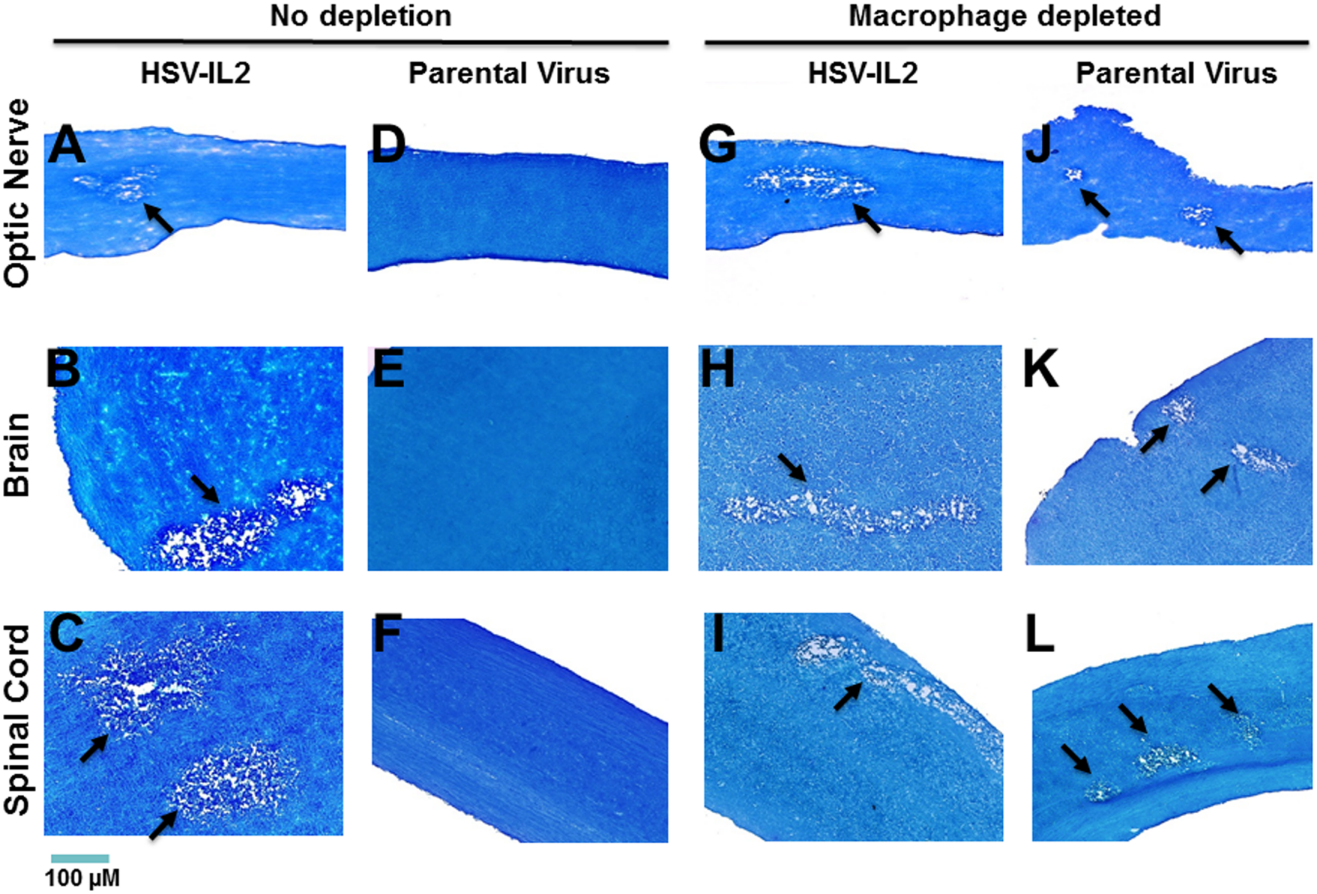
Role of macrophages in HSV-induced CNS demyelination. Female FoxP3^DTR^ mice were depleted of macrophages or mock-depleted and infected ocularly with HSV-IL-2 or parental virus (dLAT2903) (5 mice per group). Optic nerve, brain, and spinal cord were collected from euthanized mice on day 14 PI and post-fixed tissue sections were stained with LFB. Representative photomicrographs are shown (Magnification, 20_×_; Size bar, 100 μm). Arrows indicate the areas of demyelination (a plaque). Panels: A) No depletion, HSV-IL-2 infected optic nerve; B) No depletion, HSV-IL-2 infected brain; C) No depletion, HSV-IL-2 infected spinal cord; D) No depletion, parental virus infected optic nerve; E) No depletion, parental virus infected brain; F) No depletion, parental virus infected spinal cord; G) Macrophage depletion, HSV-IL-2 infected optic nerve; H) Macrophage depletion, HSV-IL-2 infected brain; I) Macrophage depletion, HSV-IL-2 infected spinal cord; J) Macrophage depletion, parental virus infected optic nerve; K) Macrophage depletion, parental virus infected brain; and L) Macrophage depletion, parental virus infected spinal cord.

To investigate the potential contribution of macrophages in the HSV-IL-2-induced CNS demyelination model, we ocularly infected FoxP3^DTR^ mice with HSV-IL-2 or parental virus in the presence and absence of macrophage depletion. As we had established that demyelination does not occur in the WT HSV-1-infected macrophage-depleted mice until after day 10 post-infection (PI) [20], we analyzed demyelination on day 14 PI. Demyelination was assessed in the optic nerve, spinal cord and brain by luxol fast blue (LFB) staining. Representative photomicrographs of optic nerve, spinal cord and brain are shown in Fig 1. We confirmed that the macrophage-depleted FoxP3^DTR^ mice that were infected with parental virus developed demyelination in the optic nerve (Fig 1A, macrophage depleted), brain (Fig 1B, macrophage depleted), and spinal cord (Fig 1C, macrophage depleted), whereas the macrophage-intact FoxP3^DTR^ mice infected with the parental virus did not develop detectable demyelination in the optic nerve (Fig 1G, no depletion), brain (Fig 1H, no depletion) or spinal cord (Fig 1I, no depletion).

The macrophage-intact FoxP3^DTR^ mice infected with HSV-IL-2 developed demyelination in the optic nerve (Fig 1J, no depletion), brain (Fig 1K, no depletion) and spinal cord (Fig 1L, no depletion), further confirming our previous findings generated using BALB/c, C57BL/6, SJL/6, and 129SVE mice. However, we found that macrophage-depleted FoxP3^DTR^ mice infected with HSV-IL-2 also developed demyelination in the optic nerve (Fig 1D, macrophage depleted), brain (Fig 1E, macrophage depleted), and spinal cord (Fig 1F, macrophage depleted). These results indicated that although macrophages can protect against WT HSV-1 infection-induced CNS demyelination, HSV-IL-2-induced demyelination occurs independently of the presence or absence of macrophages in FoxP3^DTR^ mice. In addition, the demyelinated lesions were detected as both focal and diffuse areas in the white matters of HSV-IL-2-infected mice.

### FoxP3-expressing cells mediate CNS demyelination in HSV-1-infected mice in the absence of macrophages

**Fig 2:**
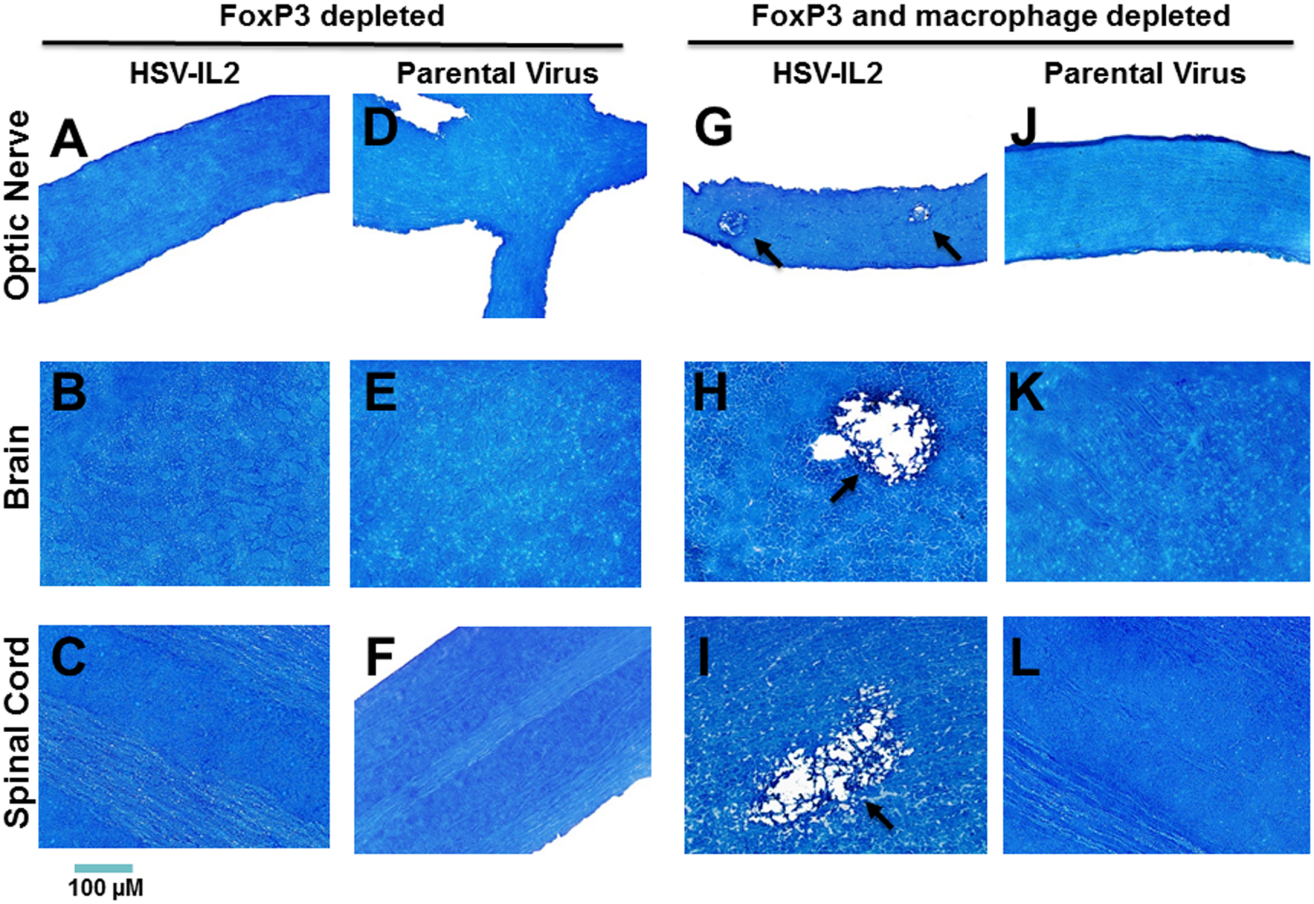
Role of FoxP3-expressing cells in HSV-induced CNS demyelination. Female FoxP3^DTR^ mice were depleted of FoxP3-expressing cells or depleted of both FoxP3-expressing cells and macrophages then ocularly infected with HSV-IL-2 or parental virus as in Fig 1. Optic nerve, brain, and spinal cord were collected from euthanized mice on day 14 PI and post-fixed tissue sections were stained with LFB. Representative photomicrographs are shown (Magnification, 20_×_; Size bar, 100 μm). Arrows indicate the areas of demyelination. Panels: A) FoxP3 depleted, HSV-IL-2 infected optic nerve; B) FoxP3 depleted, HSV-IL-2 infected brain; C) FoxP3 depleted, HSV-IL-2 infected spinal cord; D) FoxP3 depleted, parental virus infected optic nerve; E) FoxP3 depleted, parental virus infected brain; F) FoxP3 depleted, parental virus infected spinal cord; G) FoxP3 and Macrophage depleted, HSV-IL-2 infected optic nerve; H) FoxP3 and Macrophage depleted, HSV-IL-2 infected brain; I) FoxP3 and Macrophage depleted, HSV-IL-2 infected spinal cord; J) FoxP3 and Macrophage depleted, parental virus infected optic nerve; K) FoxP3 and Macrophage depleted, parental virus infected brain; and L) FoxP3 and Macrophage depleted, parental virus infected spinal cord.

IL-2 is required for the induction of Foxp3 expression and the differentiation of T_reg_ cells in the thymus [44]. We had found previously that the induction of CNS demyelination by WT HSV-1 in macrophage-depleted mice can be blocked by depletion of FoxP3-expressing cells [22]. To determine the contribution of FoxP3, macrophage-intact FoxP3^DTR^ mice were depleted of Foxp3-expressing cells and ocularly infected with HSV-IL-2 or parental virus. In this model, the depletion of Foxp3-expressing cells blocked the HSV-IL-2-induced demyelination in the optic nerve (Fig 2A, FoxP3 depleted), brain (Fig 2B, FoxP3 depleted) and spinal cord (Fig 2C, FoxP3 depleted) of infected mice. The protocol used to deplete the FoxP3-expressing cells did not contribute to demyelination as no demyelination was observed in the optic nerve (Fig 2D, FoxP3 depleted), brain (Fig 2E, FoxP3 depleted) and spinal cord (Fig 2F, FoxP3 depleted) of the macrophage-intact, FoxP3-depleted mice that were infected with parental virus. We then investigated whether FoxP3 can protect against HSV-IL-2-induced CNS demyelination in the absence of macrophages. FoxP3^DTR^ mice were depleted of both macrophages and FoxP3 prior to infection with HSV-IL-2 or parental virus. Surprisingly, HSV-IL-2 infection in the context of concomitant depletion of both FoxP3-expressing cells and macrophages resulted in demyelination in the optic nerve (Fig 2G, FoxP3 and macrophage depleted), brain (Fig 2H, FoxP3 and macrophage depleted), and spinal cord (Fig 2I, FoxP3 and macrophage depleted). Moreover, the level of demyelination was similar to that observed in FoxP3-expressing FoxP3^DTR^ mice infected with HSV-IL-2 in the absence of macrophage depletion. Replication of HSV-IL-2 in the eye of depleted mice was similar to that of parental virus. This suggests that depletion of both macrophages and FoxP3 had no direct effect on virus replication in vivo. Thus, these results indicated a complex interaction, in which the FoxP3-expressing cells contribute to blockade of CNS demyelination in HSV-IL-2 mice in the presence of macrophages, but do not block the demyelination in the absence of macrophages.

### Role of CD4^+^/CD8^+^ T cells in CNS demyelination in the absence of macrophages

**Fig 3:**
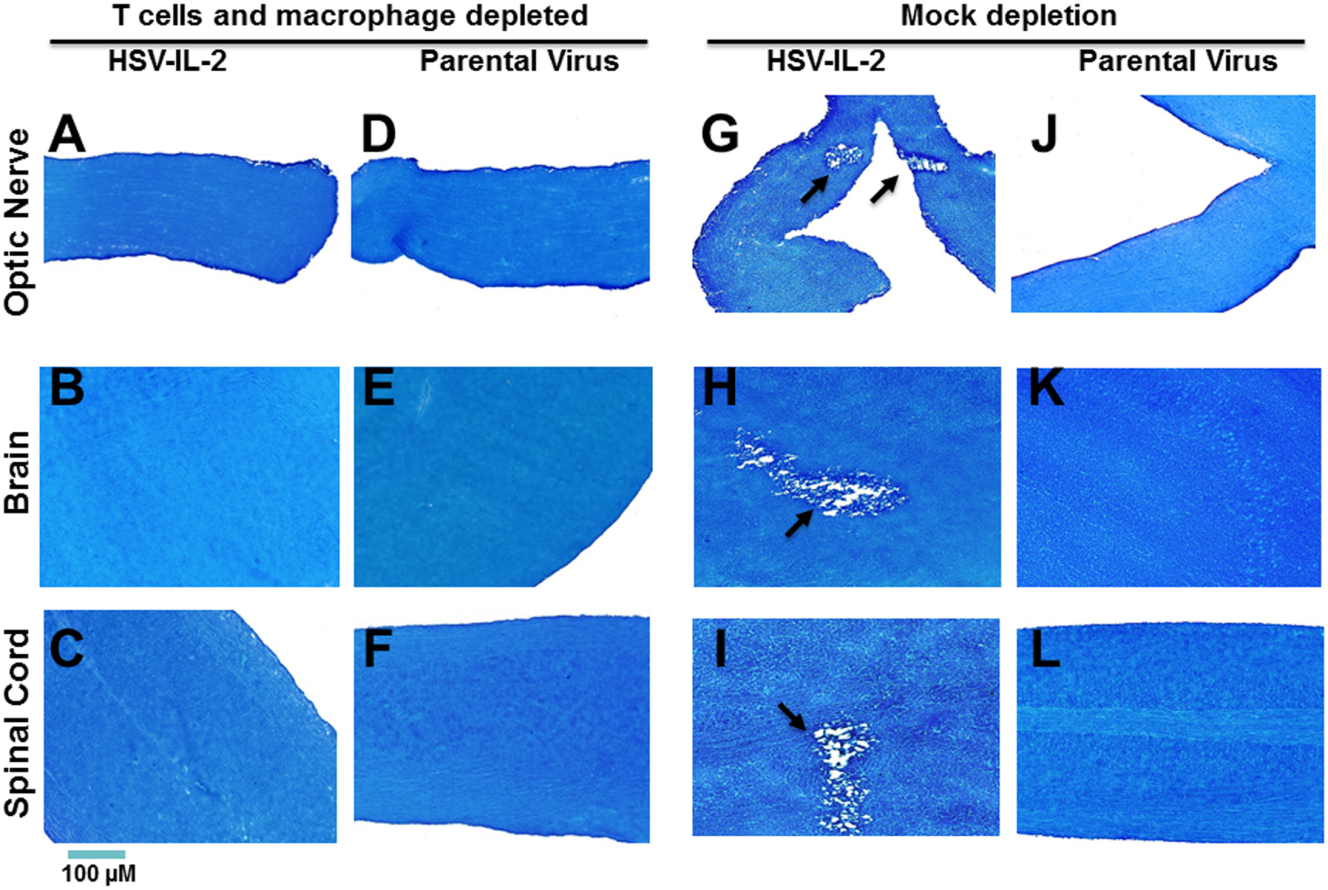
Role of T cells in HSV-induced CNS demyelination. Female WT mice were depleted of both T cells and macrophages or mock depleted then ocularly infected with HSV-IL-2 or parental virus as in Fig 1. Optic nerve, brain, and spinal cord were collected from euthanized mice on day 14 PI and post-fixed tissue sections were stained with LFB. Representative photomicrographs are shown (Magnification, 20_×_; Size bar, 100 μm). Arrows indicate the areas of demyelination. Panels: A) T cells and macrophage depleted, HSV-IL-2 infected optic nerve; B) T cells and macrophage depleted, HSV-IL-2 infected brain; C) T cells and macrophage depleted, HSV-IL-2 infected spinal cord; D) T cells and macrophage depleted, parental virus infected optic nerve; E) T cells and macrophage depleted, parental virus infected brain; F) T cells and macrophage depleted, parental virus infected spinal cord; G) Mock depletion, HSV-IL-2 infected optic nerve; H) Mock depletion, HSV-IL-2 infected brain; I) Mock depletion, HSV-IL-2 infected spinal cord; J) Mock depletion, parental virus infected optic nerve; K) Mock depletion, parental virus infected brain; and L) Mock depletion, parental virus infected spinal cord.

Previously, we found that both CD4^+^ and CD8^+^ T cells contribute to HSV-IL-2-induced CNS demyelination in macrophage intact mice [20,23] whereas in HSV-1-infected macrophage-depleted mice the demyelination can be blocked by CD4^+^ T cells alone [22]. To directly address whether T cells contribute to HSV-1-induced demyelination in the absence of macrophages, we depleted wt mice of macrophages and CD4^+^/CD8^+^ T cells or mock depleted prior to infection with HSV-IL-2 or parental virus. The HSV-IL-2 infected mice that were depleted of both macrophages and CD4^+^/CD8^+^ T cells did not show any signs of demyelination in the optic nerve (Fig 3A, T cells and macrophage depleted), brain (Fig 3B, T cells and macrophage depleted) or spinal cord (Fig 3C, T cells and macrophage depleted). Similarly, no demyelination was detected in mice infected with the parental virus that were depleted of both macrophages and CD4^+^/CD8^+^ T cells (Fig 3D, 3E and 3F, parental virus). In contrast, as we reported previously [20], demyelination was detected in mock depleted mice infected with the HSV-IL-2 (Fig 3G, 3H and 3I, mock depleted) but not in mice infected with parental virus (Fig 3J, 3K and 3L, mock depleted). These results suggest that, in the absence of macrophages, depletion of T cells can block CNS demyelination after infection with HSV-IL-2 or WT HSV-1.

### mRNA levels in the CNS of infected mice

**Fig 4:**
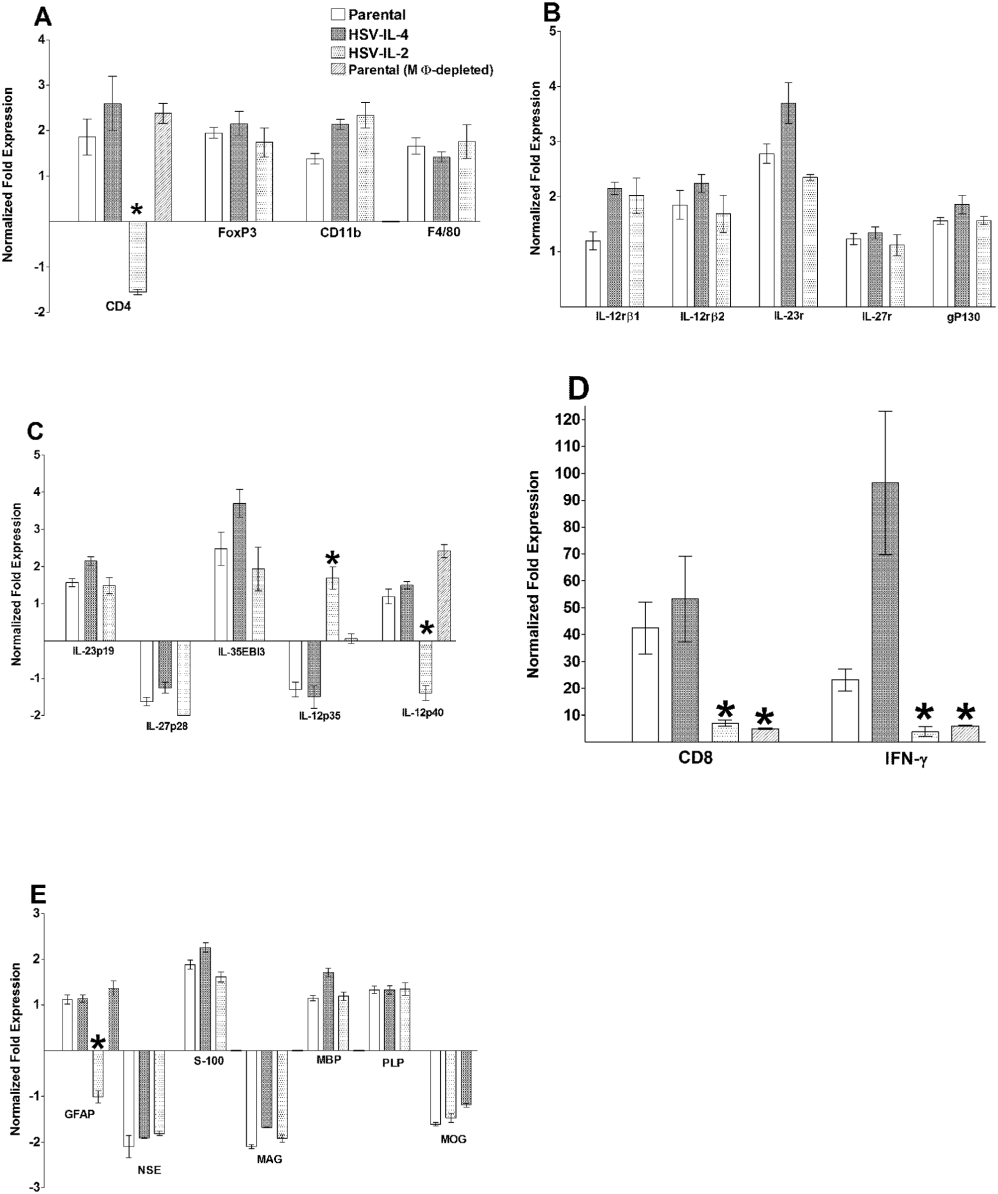
Effects of macrophage depletion on the expression of various transcripts in the brains of infected mice on day 5 PI. Female WT mice with or without macrophage depletion were ocularly infected with HSV-IL-2, HSV-IL-4, or parental virus. Brains were isolated on day 5 PI and qRT-PCR was performed using total RNA from the individual brains. GAPDH expression was used to normalize the relative expression of each transcript in the brains. The expression of each transcript in the brains of naive WT mice was determined and used to estimate the relative expression of each transcript in the brains of the ocularly infected mice. Each point represents the mean ± standard error of the mean (SEM) from 5 brains. Asterisks (*) indicate p < 0.05 by t-test. Panels: A) CD4, FoxP3, CD11b and F4/80 transcripts in brain of infected mice; B) IL12rβ1, IL12rβ2, IL23r, IL27r and gP130 transcripts in brain of infected mice; C) IL23p19, IL27p28, IL35EBI3, IL12p35, and IL12p40 transcripts in brain of infected mice; D) CD8 and IFN-γ transcripts in brain of infected mice; and E) GFAP, NSE, S-100, MAG, MBP, PLP, and MOG transcripts in brain of infected mice;

Collectively, our results supported the concept that macrophages play a major role in protection against HSV-1-induced CNS demyelination. To begin to identify the potential mechanisms involved, we compared the changes in the levels of mRNA in the brains of mice that were infected ocularly with HSV-IL-2, HSV-IL-4 or parental virus. We determined the mRNA levels of the IL-12 subunit genes (IL-23p19, IL-27p28, IL-35EBI3, IL-12p35, IL-12p40), IL-12 receptor genes (IL-12rβ1, IL-12rβ2, IL-23r, IL-27r, gp130), and markers of immune cells (CD4, CD8, FoxP3, IFN-γ, as well as CD11b and F4/80). In parallel, we determined the mRNA levels for the astrocyte marker gene, glial fibrillary acidic protein (GFAP), and demyelination marker genes (NSE, S-100, MAG, MBP, PLP, MOG). On day 5 PI, RT-PCR was performed on total RNA from individual brain. The levels of each mRNA relative to the baseline seen in the uninfected mouse brain is shown in Fig 4 and a summary of the differences between HSV-IL-2 infected and parental virus-infected mice is provided in Table 1.

**Table 1:** Comparison of mRNA expression profiles of selected genes between macrophage-intact mice infected with HSV-IL-2 or parental virus on day 5 PI^a^.

	Virus		
Transcript	HSV-IL-2	Parental	p-value
**IFN-γ**	3.8±1.8	96.51±26.7	*<0*.*0001*
**CD4**	-1.08±0.1	3.74±0.8	*<0*.*0001*
**CD8**	7.0±0.1.24	53.20±15.95	*<0*.*0001*
**IL-12p35**	1.69±0.3	-1.5±0.3	*<0*.*0001*
**IL-12p40**	-1.40±0.2	1.50±0.1	*<0*.*0001*
**GFAP**	-1.55±0.06	2.59±0.6	*<0*.*0001*

^a^Macrophage-intact female C57BL/6 mice were ocularly infected with HSV-IL-2 or parental virus. On day 5 PI, mice were euthanized and the brains were collected. Total RNA from each brain was purified and used for qRT-PCR as described in Materials and Methods. The ratio of the expression of each mRNA was normalized to its expression in the brains of uninfected control mice. The values represent the average ± SEM of the results obtained using 5 mice in each group.

The levels of CD11b and F4/80 mRNAs were similar in the macrophage-intact mice infected with HSV-IL-2, parental virus or HSV-IL-4 (Fig 4A). In terms of the IL-12-associated mRNAs, no differences were detected in the brains of mice infected with HSV-IL-2, HSV-IL-4 or parental virus groups in the mRNA levels of the IL-12 receptors (IL-12rβ1, IL-12rβ2); IL-23r, IL-27r, and gp130 (Fig 4B); or the IL-12 subunits IL-23p19, IL-27p28 and IL-35EBI3 mRNAs (Fig 4C). However, the levels of IL-12p35 mRNA, were significantly lower than baseline in the brains of mice infected with parental or HSV-IL-4 virus, but significantly higher than baseline in the IL-HSV-IL-2 infected mice (Fig 4C). Conversely, the levels of IL-12p40 mRNA were significantly higher than baseline in the parental and HSV-IL-4 infected mice but significantly lower than baseline in the HSV-IL-2 infected mice (Fig 4C). We therefore extended these experiments to analysis of the levels of IL-12p35 and IL-12p40 mRNAs in macrophage-depleted mice infected with WT HSV-1. In these mice, the levels of IL-12p35 mRNA were significantly lower than in the macrophage-intact HSV-IL-2-infected mice model but significantly higher than in the macrophage-intact mice infected with parental virus or HSV-IL-4 (Fig 4C).

In terms of the mRNA levels of T cell-associated molecules, the levels of FoxP3 mRNA were similar in the macrophage-intact mice infected with HSV-IL-2, parental virus or HSV-IL-4 (Fig 4A). However, we found that in the brains of macrophage-intact HSV-IL-2-infected mice the levels of CD4 mRNA were significantly lower than in mice infected with parental virus or HSV-IL-4 (Fig 4A) whereas the levels of CD4 mRNA in the brains of macrophage-depleted HSV-IL-2-infected mice were similar to those seen in macrophage-depleted mice infected with parental virus or HSV-IL-4 (Fig 4A). The levels of CD8 mRNA in the macrophage-intact HSV-IL-2-infected mice were significantly lower than those seen in macrophage-intact mice infected with parental virus or HSV-IL-4 (Fig 4D). In the macrophage-depleted mice infected with HSV-IL-2 virus, the levels of CD8 mRNA were similar to the levels in the macrophage-intact mice, and were significantly lower than those seen in macrophage-depleted mice infected with parental virus or HSV-IL-4 (Fig 4D). The levels of IFN-γ mRNA in the macrophage-intact HSV-IL-2 infected mice were similar to the levels in the macrophage-depleted HSV-IL-2 infected mice but significantly lower than parental virus or HSV-IL-4 infected groups, with HSV-IL-4-infected groups having the highest level of IFN-γ expression (Fig 4D).

**Fig 5:**
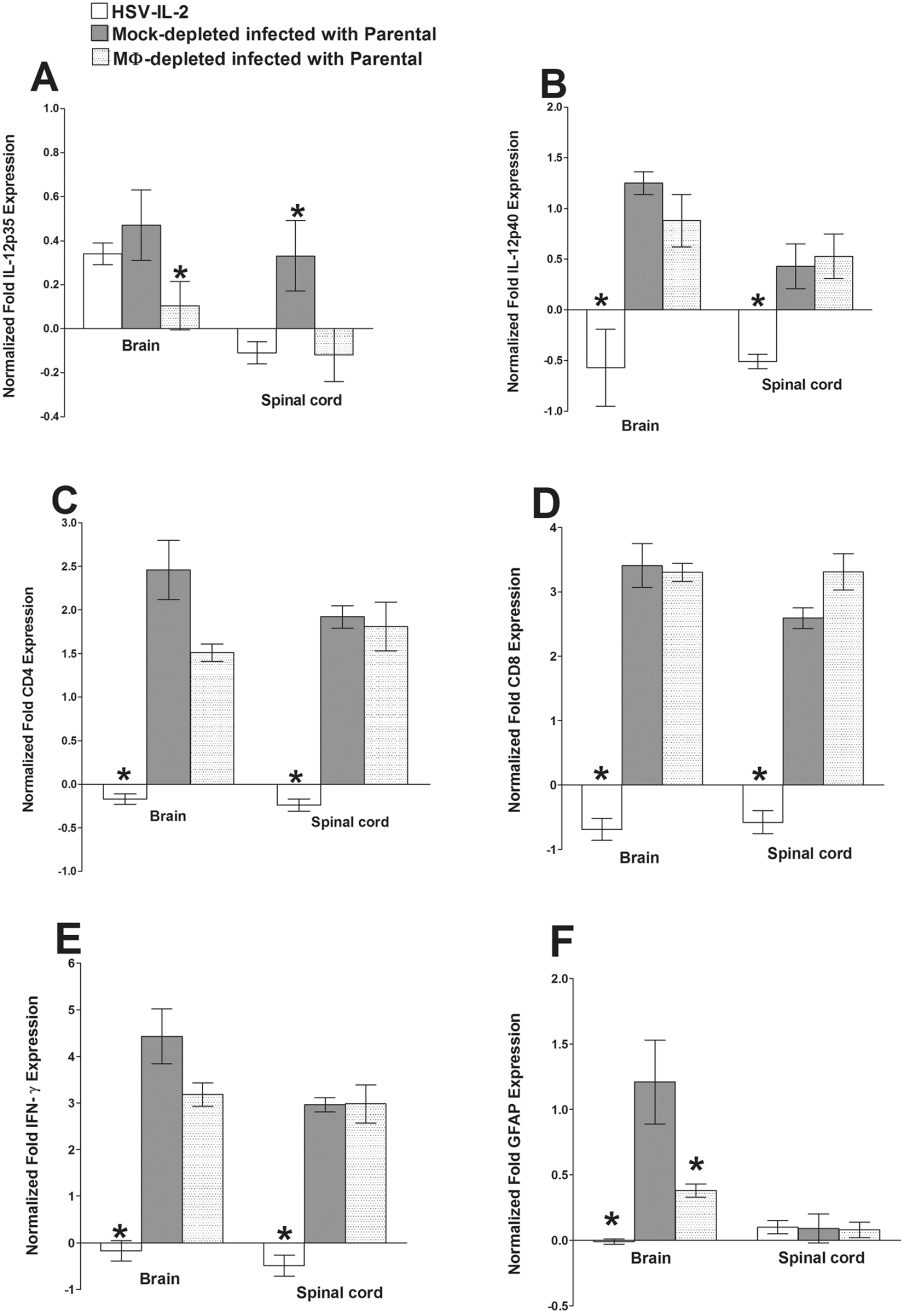
Effects of macrophage depletion on the expression of IL-12p35, IL-12p40, CD4, CD8, GFAP, and IFN-γ transcripts in brains and spinal cords of HSV-IL-2 infected mice on day 10 PI. Female WT mice with or without macrophage depletion were ocularly infected with HSV-IL-2 or parental virus as in Fig 4. The brains and spinal cords were isolated on day 10 PI and qRT-PCR was performed using total RNA extracted from the individual brains. GAPDH expression was used to normalize the relative expression of each transcript. The expression of each transcript in the brains and spinal cords of naive WT mice was determined and used to estimate the relative expression of each transcript in the brains and spinal cords of the ocularly infected mice. Each point represents the mean ± SEM from 5 brains. Asterisks (*) indicate p < 0.05 by t-test. Each point represents the mean ± SEM from 5 brains or 5 spinal cords. Asterisks (*) indicate p < 0.05 by t-test. Panels: A) IL12p35 transcript in brain and spinal cord of infected mice; B) IL12p40 transcript in brain and spinal cord of infected mice; C) CD4 transcript in brain and spinal cord of infected mice; D) CD8 transcript in brain and spinal cord of infected mice; E) IFN-γ transcript in brain and spinal cord of infected mice; and F) GFAP transcript in brain and spinal cord of infected mice.

These results suggest that the presence of IL-2 has a direct effect on the levels of IL-12p35, IL-12p40, CD4, CD8 and IFN-γ mRNAs, while depletion of macrophages affects the levels of IL-12p35, CD8, and IFN-γ mRNAs in the brain on day 5 PI. Therefore, we used the same protocol to determine the levels of these mRNAs in the spinal cord and the brain on day 10 PI in macrophage-intact mice infected with HSV-IL-2 or parental virus and macrophage-depleted mice infected with parental virus. We found that at day 10 PI, the levels of IL-12p40 mRNA in the brains of the mice infected with HSV-IL-2 or parental virus were similar and were higher than in the macrophage-depleted mice infected with parental virus (Fig 5A, brain). In contrast, the levels of IL-12p40 mRNA in the spinal cords of HSV-IL-2 infected mice were significantly lower than those in mice infected with WT parental virus and was similar to that of macrophage-depleted and infected mice (Fig 5A, spinal cord). IL-12p35 mRNA expression was suppressed in HSV-IL-2 infected mice brain compared with parental infected or macrophage-depleted mice (Fig 5B, brain), and similar patterns were observed in spinal cord of infected mice (Fig 5B, spinal cord). CD4 (Fig 5C), CD8 (Fig 5D), and IFN-γ (Fig 5E) mRNAs levels were suppressed in HSV-IL-2 infected mice brain and spinal cords compared with parental-infected or macrophage-depleted mice. In addition, the patterns of CD4 (Fig 5C), CD8 (Fig 5D), and IFN-γ (Fig 5E) mRNAs expression were similar in brain versus spinal cord of infected mice. These results for day 10 PI suggest that HSV-IL-2 has a suppressive effects on IL-12p35, IL-12p40, CD4, CD8, and IFN-γ mRNAs expression and is similar to that of their expression of day 5 PI, while macrophage depletion only affected IL-12p40 mRNA expression level. In summary, our results showed similar mRNA expression profiles for IL-12p40, CD4, CD8, IFN-γ, and GFAP but not IL-12p35 in brain and spinal cord of each group.

The levels of GFAP mRNA were significantly lower in the macrophage-intact HSV-IL-2-infected mice than macrophage-intact mice infected with HSV-IL-4 or parental virus (Fig 4E, GFAP). The levels of GFAP mRNA were similar in the macrophage-depleted mice HSV-IL-2 infected mice to that of the macrophage-depleted mice infected with HSV-IL-4 or parental virus (Fig 4E). The levels of NSE, S-100, MAG, MBP, PLP, and MOG mRNAs were similar in the brains of the HSV-IL-2, HSV-IL-4 and parental virus infected mice (Fig 4E). In the brain of infected mice, GFAP expression was the lowest for HSV-IL-2 infected mice followed by macrophage-depleted and infected mice compared with parental virus (Fig 5F, brain), while GFAP expression was similar between groups in spinal cord of infected mice (Fig 5F, spinal cord).

### HSV-IL-2-induced demyelination is blocked by transfer of macrophages infected with HSV-IL-12p40 or HSV-IL-12p70 virus but not HSV-IL-12p35 virus

The qRT-PCR studies described above suggested that an imbalance of IL-12p35 and IL-12p40 may contribute to the HSV-IL-2-induced CNS demyelination. We found previously that both HSV-IL-2-induced demyelination and the demyelination induced by WT HSV-1 in the absence of macrophages can be blocked by either IL-12p70 DNA or HSV-IL-12p70 recombinant virus [22–24]. These data raised the possibility that the IL-12p70 arm of the macrophage response plays a key role in mitigating CNS demyelination. They suggested a hypothetical model in which suppression of macrophage IL-12p35 and IL-12p40 signaling by IL-2 in the macrophage-competent HSV-IL-2 infected mice, and the lack of IL-12p70 due to macrophage depletion play a key role in the CNS demyelination in these models of MS. To test this hypothesis, we used an adoptive transfer strategy in which bone marrow (BM)-derived macrophages infected with different recombinant HSV-IL-12 viruses were transferred into recipients that were subsequently ocularly infected with HSV-IL-2. The macrophages were infected with HSV-IL-12p35, HSV-IL-12p40, HSV-IL-12p70, or parental virus, or mock-infected then were injected intravenously (*IV*) into female C57BL/6 mice. Two weeks after adoptive transfer of 1 X 10^6^ cells, the recipient mice were infected ocularly with HSV-IL-2. Fourteen days PI, the mice were sacrificed and the optic nerve, spinal cord and brain post-fixed and stained with LFB. The presence or absence of demyelination in each tissue was determined (Table 2). We found that all of the mice that received macrophages infected with HSV-IL-12p35 or WT HSV-1, or macrophages that were mock infected, developed demyelination in the optic nerve, brain or spinal cord. In marked contrast, most of the mice that received macrophages infected with HSV-IL-12p70 or HSV-1L-12p40 were protected from demyelination in the optic nerve, brain and spinal cord. The mice that received macrophages infected with HSV-IL-12p70 showed better protection than the mice that received macrophages infected with HSV-IL-12p40. In addition, the adoptive transfer of macrophages infected with HSV-IL-12p70 or HSV-1L-12p40 resulted in better protection in the optic nerve and brain of the recipient mice than in the spinal cord.

**Table 2:** Effect of adoptive transfer of macrophages on blocking CNS demyelination in HSV-IL-2 infected mice^a^.

		# of mice with demyelination / total number of recipient mice		
Cell transfer	# of Transferred cells	Optic Nerve	Brain	Spinal cord
**MΦ infected with HSV-IL-12p70**	1 X 10^6^ cells	1/5	1/5	2/5
**MΦ infected with HSV-IL-12p40**	1 X 10^6^ cells	1/5	2/5	3/5
**MΦ infected with HSV-IL-12p35**	1 X 10^6^ cells	5/5	5/5	5/5
**MΦ infected with WT HSV-1**	1 X 10^6^ cells	5/5	5/5	5/5
**Mock-infected MΦ**	1 X 10^6^ cells	5/5	5/5	5/5
**MΦ infected with HSV-IL-12p70**	1 X 10^7^ cells	0/5	0/5	0/5
**DCs infected with HSV-IL-12p70**	1 X 10^7^ cells	5/5	5/5	5/5
**Mock-infected MΦ**	1 X 10^7^ cells	5/5	5/5	5/5

^a^Bone marrow-derived macrophages treated as described in Materials and Methods were used for adoptive transfer experiments in which the specified numbers of cells were injected *i*.*v*. into recipient mice. On day 14 post transfer, the recipient mice were ocularly infected with HSV-IL-2 virus. The presence of demyelination in the optic nerve, spinal cord, and brain was assessed based on LFB staining on day 14 PI. Tissues from 5 recipient mice per group were assessed. MΦ = macrophages.

**Fig 6:**
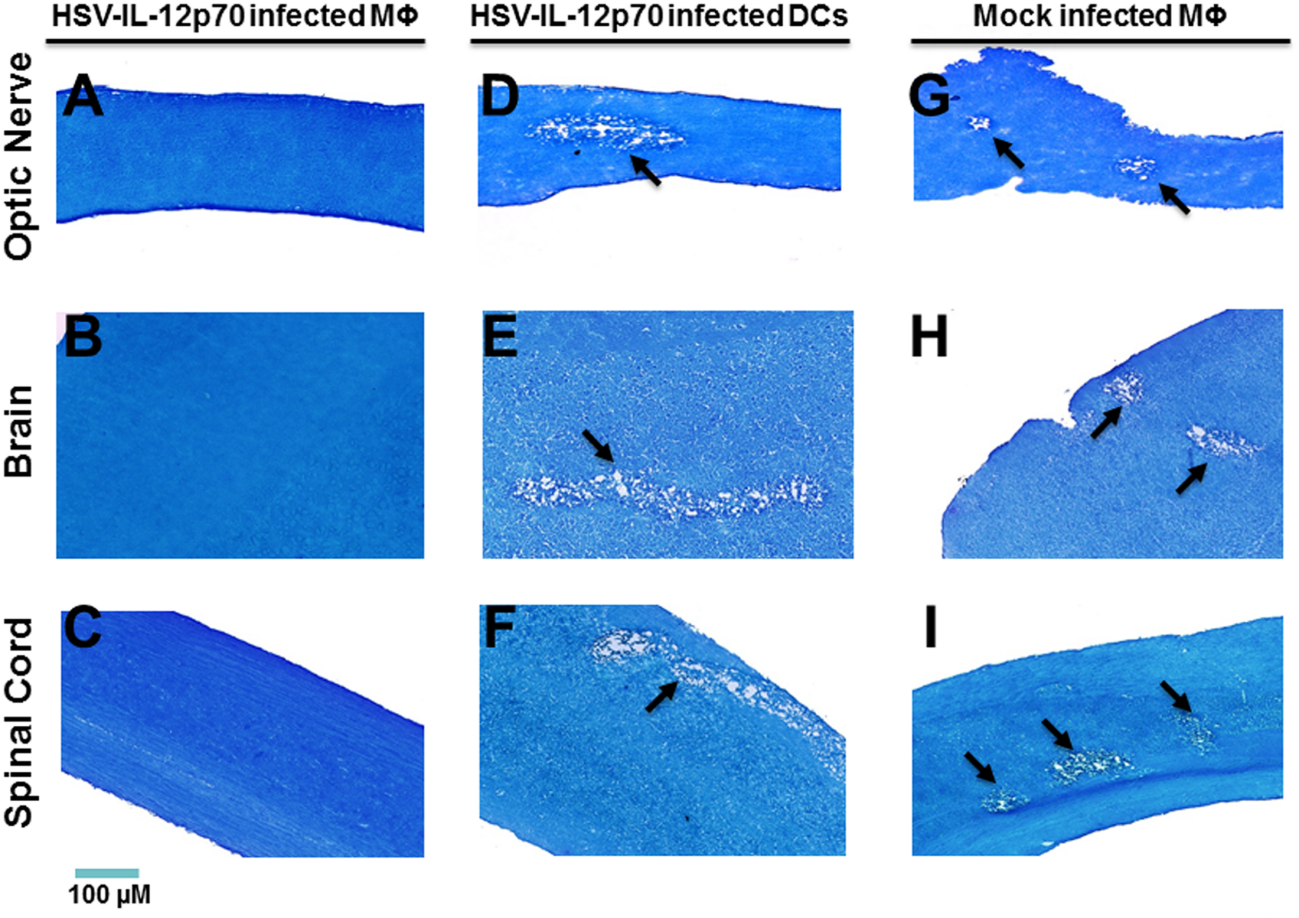
Effect of adoptive transfer of macrophages on blocking demyelination in HSV-IL-2 infected to WT mice. Bone marrow-derived macrophages or DCs from female WT mice were infected with HSV-IL-12p70 or were mock-infected. 1 X 10^6^ or 1 X 10^7^ infected or mock-infected cells were transferred *i*.*v*. into each recipient female WT mouse. Fourteen days post-adoptive transfer, recipient mice were infected ocularly with HSV-IL-2. Optic nerve, brain, and spinal cord were collected from euthanized mice on day 14 PI and post-fixed tissue sections were stained with LFB. Representative photomicrographs are shown (Magnification, 20_×_; Size bar, 100 μm). Arrows indicate the areas of demyelination. Panels: A) Effect of adoptive transfer of HSV-IL12p70 infected MΦ on demyelination in optic nerve of HSV-IL-2-infected mice; B) Effect of adoptive transfer of HSV-IL12p70 infected MΦ on demyelination in brain of HSV-IL-2-infected mice; C) Effect of adoptive transfer of HSV-IL12p70 infected MΦ on demyelination in spinal cord of HSV-IL-2-infected mice; D) Effect of adoptive transfer of HSV-IL12p70 infected DCs on demyelination in optic nerve of HSV-IL-2-infected mice; E) Effect of adoptive transfer of HSV-IL12p70 infected DCs on demyelination in brain of HSV-IL-2-infected mice; F) Effect of adoptive transfer of HSV-IL12p70 infected DCs on demyelination in spinal cord of HSV-IL-2-infected mice; G) Effect of adoptive transfer of mock infected MΦ on demyelination in optic nerve of HSV-IL-2-infected mice; H) Effect of adoptive transfer of mock infected MΦ on demyelination in brain of HSV-IL-2-infected mice; and I) Effect of adoptive transfer of mock infected MΦ on demyelination in spinal cord of HSV-IL-2-infected mice.

We then repeated the experiment using a higher dose (1 X 10^7^) macrophages infected with HSV-IL-12p70 or HSV-IL-12p40 and, as control, transfer of 1 X 10^7^ DCs infected with HSV-IL-12p70. Representative photomicrographs are shown in Fig 6 and a summary of the results is provided in Table 2. No demyelination was detected in optic nerve, brain and spinal cord sections of mice that received 1 X 10^7^ macrophages infected with HSV-IL-12p70 (Fig 6, Table 2) or HSV-IL-12p40, whereas demyelination occurred in the mice that received HSV-IL-12p70-infected DCs or mock-infected macrophages prior to infection (Fig 6, Table 2).

**Fig 7:**
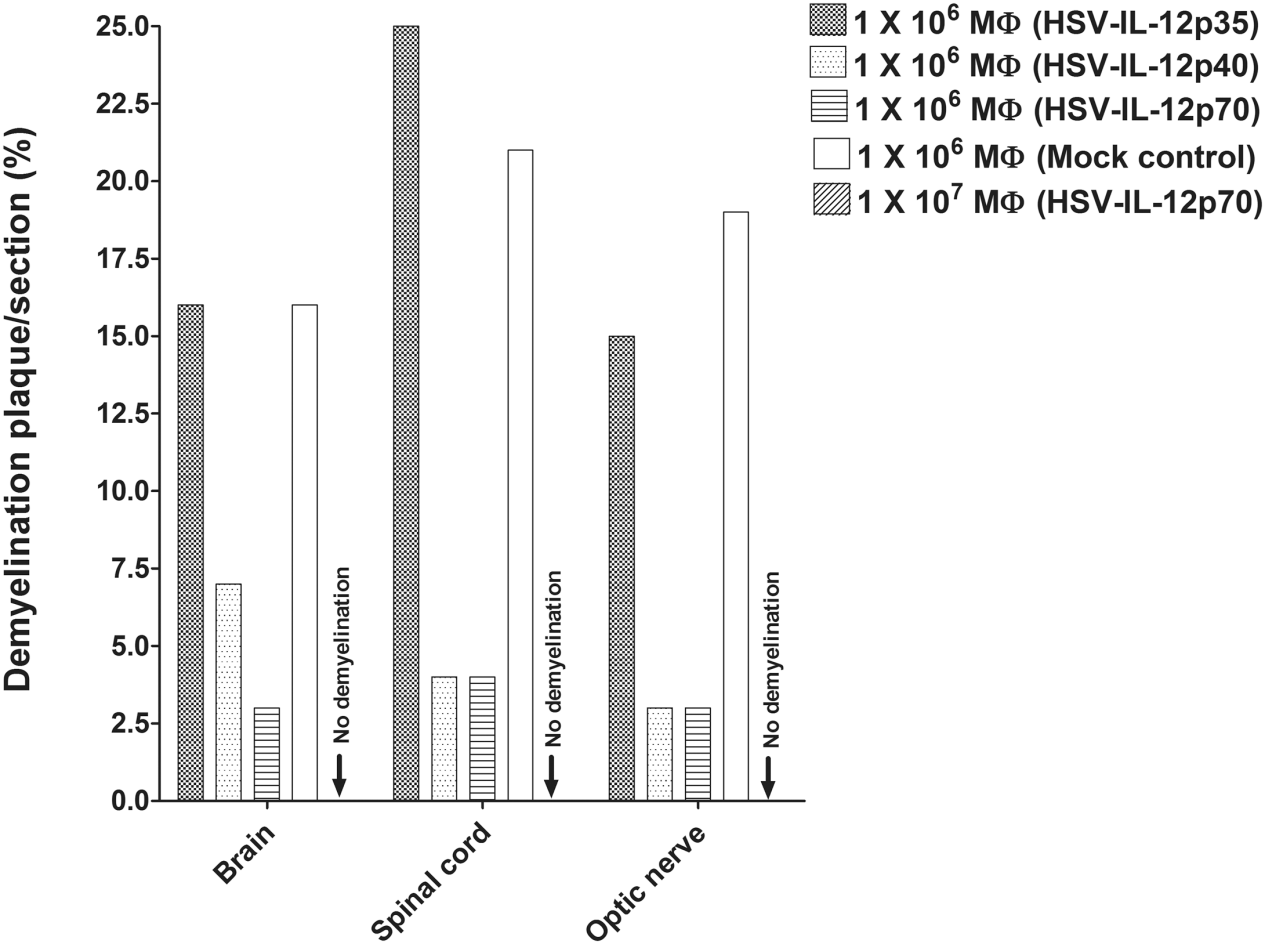
Severity of CNS demyelination in macrophage recipient mice. The LFB-stained sections of the brains, spinal cords, and optic nerves of WT mice that received adoptively transferred macrophages from WT mice and analyzed as described in Table 2 were further analyzed in terms of the size of the demyelination plaques in the entire sections of brain, spinal cord, and optic nerves. Data are presented as mean demyelination using a total of 150 sections for brain and spinal cord and 30 sections for optic nerve from 5 mice per group. Arrows indicate no demyelination in the brain, spinal cord and optic nerve of mice received 1 X 10^7^ macrophages infected with HSV-IL-12p70 virus.

Image analysis of the stained tissue sections suggested that the extent of demyelination differed amongst the experimental groups and the CNS tissue. In those mice that received 1 X 10^6^ macrophages, the area of demyelination in the brains of mice was significantly larger in the mice that received HSV-IL-12p35-infected macrophages or mock-infected macrophages than the area of demyelination in the mice that received HSV-IL-12p40- or HSV-IL-12p70-infected macrophages (Fig 7, Brain). Moreover, the area of demyelination in the brains of mice that received HSV-IL-12p70-infected macrophages was lower than the area of demyelination in the brains of mice that received HSV-IL-12p40-infected macrophages (Fig 7, Brain). As described above, no demyelination was detected in brains of mice that received 1 X 10^7^ HSV-IL-12p70-infected macrophages (Fig 7, Brain, Arrow: no demyelination). In those mice that received 1 X 10^6^ macrophages, the area of demyelination was somewhat higher in the spinal cords of mice that received HSV-IL-12p35-infected macrophages than the area of demyelination in the spinal cord of mice that received mock-infected macrophages (Fig 7, Spinal cord). The level of demyelination in the spinal cord of mice that received HSV-IL-12p40-infected macrophages were similar to the level of demyelination in the spinal cord in mice that received HSV-IL-12p70-infected macrophages (Fig 7, Spinal cord). In contrast to the spinal cords, the area of demyelination in the optic nerves was somewhat lower in recipient mice that received HSV-IL-12p35-infected macrophages than the area of demyelination in the optic nerve of mice that received mock-infected macrophages (Fig 7, Optic nerve). However, the level of demyelination in the optic nerves of mice that received HSV-IL-12p40-infected virus were similar to the levels of demyelination in the optic nerves of mice that received HSV-IL-12p70-infected macrophages (Fig 7, Optic nerve). As described above, no demyelination was detected in spinal cord and optic nerve of mice that received 1 X 10^7^ macrophages infected with HSV-IL-12p70 virus (Fig 7, Spinal cord, Optic nerve, Arrow: no demyelination).

## Discussion

We reported previously that CNS demyelination occurred following ocular infection of mice with HSV-IL-2 virus, while WT viruses, HSV-IFN-γ or HSV-IL-4 did not induce CNS demyelination [19,20,22–24]. In addition, severity of CNS demyelination in HSV-IL-2-infected mice was sex-dependent [20]. Similar results were reported for MS patients [45] and EAE model of MS [46]. Thus, in this study we used female mice for all our experiments. We also have shown that macrophages, but not B cells, DCs, NK cells or T cells, mediated self-tolerance and protection against autoimmunity following ocular infection with WT HSV-1 in a manner similar to that of HSV-IL-2 in HSV-1-infected mice [22–24]. Previously we have shown that macrophages play a significant role in blocking CND demyelination in mice infected ocularly with WT HSV-1 [22].

In the current study, we show an imbalance of IL-12p35 and IL-12p40 mRNA levels in the CNS of macrophage-intact HSV-IL-2-infected mice on days 5 and 10 PI that does not occur in parental virus-infected mice. A similar imbalance was observed in macrophage-depleted mice infected with parental virus. The results suggested that this effect was specific for these IL-12 subunits, as the transcription of other members of the IL-12 family that are involved in formation of IL-23, IL-27, and IL-35 were not affected. These results are is similar with our previous findings that IL-12p35^–/–^and IL-12p40^–/–^mice developed CNS pathology following ocular HSV-1 infection with WT viruses and that this demyelination did not occur when each knockout strain was reconstituted with its missing gene [22]. Moreover, this pathology was not detected in HSV-1-infected IL-23p19^–/–^mice or in EBI3^–/–^mice. Our previous data and the present study suggest that both p35 and p40 subunits of IL-12 are required for protection from CNS demyelination. Although the accumulation of macrophages around demyelination plaques suggests that they may play a pathologic role [47], it is possible that their accumulation simply reflects their phagocytic function. Our current results demonstrate that macrophages, through their production of IL-12p70, play a central role in protection from virus-induced demyelinating immunopathology.

Injection of mice with IL-12p70 DNA prevented development of CNS demyelination in macrophage-depleted mice. However, IL-12p35 or IL-12p40 DNA alone, or together had no protective effect on prevention of CNS demyelination in WT macrophage-depleted mice, indicating that control of CNS demyelination is dependent on the IL-12p70 heterodimer [22]. Similarly, we have shown previously that demyelination induced by HSV-IL-2 can be blocked by either injection of IL-12p70 DNA or a recombinant HSV-1 expressing IL-12p70 [23,24]. In the current study, we have shown that transfer of macrophages infected with HSV-1 recombinant virus expressing IL-12p70 or IL-12p40, but not IL-12p35 protected HSV-IL-2 infected mice from CNS demyelination in a dose-dependent manner. Higher demyelination in macrophages infected with IL-12p35 may be due to higher expression of IL-7 by microglia and macrophages. Previously it was reported that IL-12p35, but not IL-12p40, subunit of IL-12p70 is involved in the induction of IL-7 in microglia and macrophages [48]. Furthermore, increase of IL-7 expression were reported for individual with MS and in EAE model of MS [49,50]. In contrast, DCs infected with IL-12p70 or mock-infected macrophages did not block demyelination. Previously, we have shown that both macrophages and DCs can be infected with HSV-1 but the virus does not replicate and does not increase apoptosis or cell death in infected macrophages or DCs [51]. Thus, our results suggest that macrophages carrying IL-12p70, but not DCs or macrophages without IL-12p70, can compensate for the suppressive effects of IL-2 on the IL-12p70 components.

In the current study, we found that the effects of depletion of FoxP3-expressing cells on demyelination was highly dependent on the experimental model. In HSV-IL-2-infected mice, depletion of FoxP3-expressing cells blocked demyelination in mice, whereas depletion of macrophages as well as FoxP3-expressing cells did not block demyelination. In macrophage-depleted parental HSV-1-infected mice, demyelination was blocked following depletion of FoxP3-expressing cells. Previously, we had found that in WT HSV-1-infected mice, the absence of CD25 also blocked demyelination in macrophage-depleted mice [22] but not in the HSV-IL-2 model of demyelination [23]. These results are consistent with the reports by other investigators that depletion of T_reg_ cells can result in enhanced immune responses against some infectious agents [52] and that T_reg_ cells can enhance tissue damage and autoimmunity [53–58]. The reports that IL-2 can expand and induce T_reg_ cells *in vivo* [59] and *in vitro* [60], are in line with our present study showing that depletion of FoxP3-expressing cells blocked CNS demyelination by HSV-IL-2 and required both IL-2 and viral infection. Thus, IL-2 can modulate effector and T_reg_ cell function in the presence of HSV-1 infection. However, the absence of demyelination in the mice that were depleted of FoxP3-expressing cells infected with HSV-IL-2 was dependent on the presence of macrophages. In the mice that were depleted of FoxP3-expressing cells and macrophages and infected with HSV-IL-2, the depletion of both CD4 and CD8 was required for blocking demyelination independent of CD25. In contrast, as we reported previously demyelination in the absence of macrophages in mice infected with WT virus can be blocked by the absence of FoxP3, CD4, or CD25 [22]. In the HSV-IL-2 infected mice that were depleted of their macrophages but not in macrophage depleted mice infected with parental virus, the level of both CD4 and CD8 expression were reduced significantly. Thus, IL-2 signaling may be involved with contraction of T-cell responses in the HSV-IL-2 infected mice. Previously, it was shown that IL-2 signaling enhances susceptibility of T cells to apoptosis [61]. In addition, IL-2 impairs T follicular helper (T_fh_) cells [62], while enhancing induced T_reg_ (iT_reg_) [60].

We found previously that between days 3 and 7 PI, HSV-IL-2-infected mice exhibit a mixed T_H_1 + T_H_2 response, whereas mice infected with HSV-IFN-γ exhibit a T_H_1 response [19]. Similarly, we have shown that mice infected with HSV-IL-2 had an imbalance of T_H_1/T_c_1 cytokines as compared with WT HSV-1 or recombinant viruses expressing IL-4 or IFN-γ [19]. In the present study, the levels of IFN-γ were significantly reduced in the brain of HSV-IL-2 infected mice as well as in macrophage-depleted mice. Thus, these data suggest that suppression of IL-12p70 formation by combination of IL-2 and HSV-1 infection shifts the immune response from a T_H_1 response, which could promote T cell autoreactivity and induction of demyelination. Surprisingly, the levels of both CD4^+^ and CD8^+^ T cells in HSV-IL-2 infected mice were reduced as compared with parental and HSV-IL-4 viruses. This reduction of T cells in the CNS of HSV-IL-2 infected mice may have been accompanied by a skewing of the population from protective T cells to pathogenic T cells. With regards to macrophage-depleted and HSV-1 infected mice, we found that depletion of macrophages affected the CD8^+^ but not CD4^+^ T cells. Thus, this imbalance of T cells also may be responsible for generation of pathogenic CD4^+^ T cells in macrophage-depleted mice that were infected with WT HSV-1.

Previously, we reported that depletion of macrophages enhanced infection of GFAP^+^ astrocytes in the spinal cords of HSV-1 infected mice as compared to mock-depleted mice [22]. Image analyses of HSV-1-infected mice revealed a significantly higher GFAP burden in the spinal cord white matter and grey matter of macrophage-depleted *vs*. mock-depleted mice. In contrast, in the present study we observed a significant suppression of GFAP mRNA expression in the brains of HSV-IL-2-infected mice but not in the brains of other groups. The suppression of GFAP mRNA in the HSV-IL-2-infected mice on day 5 PI suggests that the astrocytes are not activated. However, by day 10 post infection GFAP mRNA expression was significantly lower in the brains of HSV-IL-2-infected and macrophage-depleted mice compared with parental virus, while its expression in the spinal cords of all group was similar but lower than on day 5 PI. The discrepancy, between our two models of demyelination with regards to the level of GFAP mRNA expression could be due the presence of IL-2 in our HSV-IL-2 model of demyelination. Similar to the results of our current study, varicella zoster virus (VZV) infection has been shown to downregulate GFAP mRNA expression *in vitro* [63]. Additionally, it was reported that loss of astrocytes occurs before that of CNS demyelination [64]. In this report, we demonstrate that GFAP expression was significantly affected by HSV-IL-2 infection or after depletion of macrophages and infection with WT HSV-1. This suggests that a relationship exists between astrocytes and IL-2 or astrocytes and macrophages that control CNS pathology. Our results are supported by recent evidence that interruption of astrocyte function exacerbates pathogenesis of CNS diseases [65]. We propose that suppression of GFAP on astrocytes in the absence of macrophages or in the presence of IL-2 following infection with HSV-1 affect IL-12p70 expression thus leading to autoreactivity of T cells and thus CNS demyelination. However, this suppressive effect can be reversed by IL-12p70 or IL-12p40. In contrast to this study, IL-2 treatment has been reported to increase GFAP expression and induce inflammation and macrophage infiltration [66]. The discrepancy between our results and this study is probably due to the HSV-1 infection in our study.

Despite the presence of demyelinated plaques in the CNS of HSV-IL-2 infected mice, no significant change was observed in the mRNA levels of the demyelination marker genes (NSE, S100, MAG, MBP, PLP, MOG). These results suggest that the effects of HSV-IL-2 on demyelination are not executed at the transcriptional level. Any of these genes alone or in combination have been associated with degradation of myelin by activated T cells in the CNS of infected mice. Recently, we compared MOG_35–55_, MBP_35–47_, and PLP_190–209_ induced models of EAE with our HSV-IL-2-induced MS model [67]. CNS pathology in MOG treated mice was similar to that of HSV-IL-2 treated mice but both were different from MBP or PLP injected mice. The similarity of our HSV-IL-2 model of demyelination to the MOG-induced model of demyelination may suggest that HSV-IL-2 autoreactive T cells affect the MOG component of myelin. However, the contributions of other members of myelin, such as MBP and PLP alone or in combination, to CNS demyelination in our model cannot be ruled out.

In summary, our results suggest that suppression of IL-12p70 causing the FoxP3^+^ T cells to become autoreactive leading to demyelination of the CNS in the infected mice. However, in contrast to our study, previous study found that the absence of the Foxp3^+^ T cells causing autoimmunity in both humans and mice [68–70]. We feel that these contrasting results most likely stem from the infection of HSV-1 in the presence of IL-2 over-expression or in the absence of macrophages in our two models of MS.

## Materials and methods

### Ethics statement

All animal procedures were performed in strict accordance with the Association for Research in Vision and Ophthalmology Statement for the Use of Animals in Ophthalmic and Vision Research and the NIH *Guide for the Care and Use of Laboratory Animals* (ISBN 0-309-05377-3). Animal research protocol was approved by the Institutional Animal Care and Use Committee of Cedars-Sinai Medical Center (Protocols #2841 and 6134).

### Viruses and mice

Plaque-purified HSV-IL-2, HSV-IL-4, dLAT2903, HSV-IL-12p35, HSV-IL-12p40, and HSV-IL-12p70 were grown in rabbit skin (RS) cell monolayers in minimal essential medium (MEM) containing 5% fetal calf serum (FCS) as we described previously [19,20,22–24,71,72]. dLAT2903 is the parental virus for HSV-IL-2, HSV-IL-4, HSV-IL-12p35, and HSV-IL-12p40 and is referred to as parental virus. Female C57BL/6 mice of 6 weeks of age were purchased from the Jackson Laboratory (Bar Harbor, ME). C57BL/6-FoxP3^DTR^ mice were a gift from Alexander Y. Rudensky (Memorial Sloan Kettering Cancer Center, New York) and were bred in the Animal Facility at the Cedars-Sinai Medical Center and we only used female C57BL/6-FoxP3^DTR^ mice for this study.

### Ocular infection

As we described previously [19,20,22–24,71,72], mice were infected ocularly in both eyes with 2 x 10^5^ PFU per eye for each virus. Each virus was resuspended in 2 μl of tissue culture media and administered as an eye drop. No corneal scarification was performed prior to infection. No behavioral changes were observed between infected animals.

### Macrophage depletion

Liposome-encapsulation of dichloromethylene diphosphonate (Cl_2_MDP) was purchased (ClodronateLiposomes.org, Netherland) and depletions were carried out as we described previously [22,73]. Briefly, mice were injected twice with 100 μl of the mixture, once intraperitoneally (*i*.*p*.) and once subcutaneously (*s*.*c*.), on days -5, -2, +1, +4, and +7 relative to ocular infection with HSV-1.

### Tissue preparation

Optic nerves, brains, and spinal cords of experimental and control mice were removed at necropsy on day 14 PI, embedded in OCT (Tissue-Tek, Sakura Finetek, Torrance, CA) for cryosectioning, and stored at -80°C as we described previously [20].

### Analysis of demyelination using luxol fast blue (LFB) staining

Transverse sections of ONs, brains, and SCs, 10 μm thick (spaced 50μm apart), were prepared using a Leica CM3050S cryostat, air-dried overnight, and fixed in acetone for 3 min at 25°C [74]. The presence or absence of demyelination in infected mice was evaluated using LFB (Sigma-Aldrich) staining of formalin-fixed sections of CNS as we described previously [19,20,22–24]. Demyelination in each section was confirmed by monitoring adjacent sections.

### Depletion of FoxP3

Female C57BL/6-FoxP3^DTR^ mice were depleted of FoxP3 by treatment with diphtheria toxin (DT) (Sigma-Aldrich, Saint Louis, MO) as described previously [22,75]. Briefly, the mice were administered DT at 72 and 24 h before ocular infection, followed by four additional treatments on days +1, +3, +5, and +7 PI. Efficiency of FoxP3 depletion in spleens were monitored by flow cytometry analysis before ocular infection and 7 days after ocular infection. After three depletions, more than 97% of FoxP3^+^ T cells were depleted.

### Depletion of T cells

Each mouse received an *i*.*p*. injection of 100 μg of purified GK1.5 (anti-CD4) and 100 μg of 2.43 (anti-CD8) monoclonal antibodies (NCCC, Minneapolis, MN) in 100 μl of PBS, -5 and -2 days before ocular infection as we described previously [23]. The injections were then repeated on days +1, +4, +7, and +10 relative to ocular infection. Control mice were depleted using an irrelevant monoclonal antibody of the same isotype. The efficiency of CD4^+^ and CD8^+^ T-cell depletion was monitored by flow cytometry of splenocytes 24 h after the second depletion and before ocular infection. After the second depletion, more than 95% of CD4^+^ T cells and CD8^+^ T cells were depleted from spleen.

### RNA extraction, cDNA synthesis, and TaqMan qRT-PCR assay

Brain and spinal cord from individual mice were collected on day 5 or 10 PI, immersed in RNAlater RNA stabilization reagent (Qiagen, Valencia, CA) and stored at -80°C until processing as we described previously [73,76]. The mRNA expression levels of IL-23p19, IL-27p28, IL-35EBI3, IL-12p35, IL-12p40, GFAP, NSE, S-100, MAG, MBP, PLP, MOG, IL-12rβ1, IL-12rβ2, IL-23r, IL-27r, gp130, CD4, FoxP3, CD11b, F4/80, CD8, and IFN-γ were determined using commercially available TaqMan Gene Expression assays (Applied Biosystems) with optimized primers as described below. In all experiments, GAPDH was used for normalization of transcripts. In this study we looked at the mRNA expression level and not protein expression.

Primer probe sets consisted of two unlabeled PCR primers and the FAM dye-labeled TaqMan MGB probe formulated into a single mixture. All cellular amplicons included an intron-exon junction to eliminate signal from genomic DNA contamination. The assays used in this study were as follows: 1) IL-23 p19, ABI assay I.D. Mm00518984_m1—amplicon length = 61 bp, 2) IL-27 p28, ABI assay I.D. Mm00461164_m1—amplicon length = 69 bp, 3) IL-35 Ebi3, ABI assay I.D. Mm00469294_m1—amplicon length = 123 bp, 4) IL-12 p35, ABI assay I.D. Mm00434165_m1—amplicon length = 68 bp, 5) IL-12 p40, ABI assay I.D. Mm01288990_m1—amplicon length = 105 bp, 6) GFAP, ABI assay I.D. Mm01253033_m1—amplicon length = 75 bp, 7) NSE, ABI assay I.D. Mm00469062_m1—amplicon length = 76 bp, 8) S-100, Mm00485897_m1—amplicon length = 69 bp, 8) MAG, ABI assay I.D. Mm00487538_m1—amplicon length = 94 bp, 9) MBP, ABI assay I.D Mm01262035_m1—amplicon length = 83 bp, 10) PLP, ABI assay I.D. Mm00456892_m1—amplicon length = 67 bp, 11) MOG, ABI assay I.D. Mm00447824_m1—amplicon length = 93 bp, 12) IL-12rβ1, ABI assay I.D. Mm00434189_m1—amplicon length = 60 bp, 13) IL-12rβ2, ABI assay I.D. Mm00434200_m1—amplicon length = 74 bp, 14) IL-23r, ABI assay I.D. Mm00519943_m1—amplicon length = 72 bp, 15) IL-27r, ABI assay I.D. Mm00497259_m1—amplicon length = 69 bp, 16) gp130, ABI assay I.D. Mm00439668_m1—amplicon length = 89 bp, 17) CD4, ABI assay I.D. Mm00442754_m1—amplicon length = 78 bp,18) FoxP3, ABI assay I.D. Mm00475164_m1—amplicon length = 80 bp, 19) CD11b, ABI assay I.D. Mm00434455_m1—amplicon length = 69 bp, 20) F4/80, ABI assay I.D. Mm00802529_m1—amplicon length = 92 bp, 21) CD8, ABI assay I.D. Mm01182108_m1—amplicon length = 67 bp, 22) IFN-γ, ABI assay I.D. Mm00801778_m1—amplicon length = 101 bp, and 23) GAPDH, ABI assay I.D. Mm999999.15_G1 –amplicon length = 107 bp. Additionally, a custom-made primer and probe set was used for LAT as follows: forward primer, 5'-GGGTGGGCTCGTGTTACAG-3'; reverse primer, 5'-GGACGGGTAAGTAACAGAGTCTCTA-3'; and probe, 5'- FAM-ACACCAGCCCGTTCTTT-3'–Amplicon Length = 81 bp. Quantitative real-time PCR (qRT-PCR) was performed using an ABI ViiA 7 Sequence Detection System (Applied Biosystems) in 384-well plates as we described previously [73,76].

### *In vitro* culture of macrophages and DCs

Six-week-old female C57BL/6 mice were used as a source of bone marrow (BM) for the generation of mouse DCs and macrophages in culture as we described previously [51]. Briefly, BM cells were isolated by flushing femurs and tibiae with PBS. Pelleted cells were resuspended briefly in water to lyse red blood cells and stabilized by adding complete medium (RPMI 1640, 10% fetal bovine serum, 100 U/ml penicillin, 100 μg/ml streptomycin, 2 mM L-glutamine). The cells were centrifuged and resuspended in complete medium supplemented with murine CSF1 (100 ng/ml; Peprotech, Rocky Hill, NJ) to grow macrophages, while to grow DCs the media was supplemented with murine GM-CSF (100 ng/ml; Peprotech). The cells were plated in non-tissue culture plastic petri dishes (1 bone per 10 cm dish) for 5 d at 37°C with CO_2_. After 5 d, the media was removed, and the adherent cells recovered by incubating the cells for 5 min at 37°C with Versene (Invitrogen, San Diego, CA). Cells were washed, counted, and plated onto tissue culture dishes for use the following day.

### Infection of macrophages or DCs *in vitro*

Monolayers of macrophages were infected with 1 PFU/cell of dLAT2903, HSV-IL-12p35, HSV-IL-12p40, or HSV-IL-12p70, and monolayers of DCs were infected with 1 PFU/cell of HSV-IL-12p70. One hour after infection at 37°C, virus was removed and the infected cells were washed three times and fresh media was added to each well. The monolayers at 24 h PI were harvested, washed, and counted for subsequent adoptive transfer experiments.

### Adoptive transfer

Each recipient female C57BL/6 mouse was injected once intravenously (*i*.*v*.) with 1 X 10^6^ or 1 X 10^7^ infected macrophages in 100 μl of MEM. Similarly control mice received uninfected macrophages or infected DCs. Recipient mice were ocularly infected two weeks after transfer with HSV-IL-2 virus.

### Measurement of extent of demyelination

The numbers of plaques and size of plaques on multiple LFB stained fields were evaluated in a blind fashion for each treatment group by inspection of serial sections of CNS tissues. The amount of myelin loss in the stained sections of brains, SCs and ONs was measured using NIH Image J software as described previously [67]. The areas of demyelination (clear-white) to normal tissue (blue) were quantified using 75 random sections from the brain and SCs or 30 sections from ONs of each animal. Demyelination in each section was confirmed by monitoring adjacent sections. The percentage of myelin loss was calculated by dividing the lesion size by the total area for each section.

### Statistical analyses

Level of demyelination in experimental and control groups were compared using Fisher’s exact tests. Student’s t test was performed for comparison of means of differences using Instat (GraphPad, San Diego). Results were considered statistically significant when the "P" value was <0.05.
